# Phaeochromocytoma Crisis: Two Cases of Undiagnosed Phaeochromocytoma Presenting after Elective Nonrelated Surgical Procedures

**DOI:** 10.1155/2013/514714

**Published:** 2013-10-28

**Authors:** P. C. Johnston, J. A. Silversides, H. Wallace, P. A. Farling, A. Hutchinson, S. J. Hunter, F. Eatock, K. R. Mullan

**Affiliations:** ^1^Regional Centre for Endocrinology and Diabetes, Royal Victoria Hospital, Grosvenor Road, Belfast BT12 6BA, UK; ^2^Regional Intensive Care Unit, Royal Victoria Hospital, Belfast, UK; ^3^Department of Anaesthesia, Royal Victoria Hospital, Belfast, UK; ^4^Department of Endocrine Surgery, Royal Victoria Hospital, Belfast, UK

## Abstract

Phaeochromocytoma is a catecholamine producing tumour and an uncommon cause of hypertension. We present two cases of relatively asymptomatic individuals, in which previously undiagnosed phaeochromocytoma was unmasked by elective nonadrenal surgical procedures, manifesting as postoperative hypertensive crisis and subsequent cardiogenic shock. The initial management in intensive care is discussed, in addition to the clinical and biochemical diagnostic challenges present. Successful adrenalectomy was performed in each case.

## 1. Introduction

Phaeochromocytomas are rare catecholamine secreting tumours arising from chromaffin cells of the adrenal medulla and extra adrenal paragangliomas [1]. Secretion of catecholamines in phaeochromocytoma can be episodic or biochemically silent and therefore can present difficulty in diagnosis; however, failure to diagnose phaeochromocytoma can have fatal consequences. Classic symptoms include headache, palpitations, sweats, and sustained or paroxysmal hypertension, as a result of elevated catecholamine production [2]; however, presentation can be varied. A “hypertensive crisis” as the initial manifestation of undiagnosed phaeochromocytoma is rare and can have a high mortality rate. Although presentation in the perioperative setting in the context of a nonadrenal surgical procedure is infrequent, anaesthetists, intensivists, surgeons, and others who care for such patients must have an awareness of this condition in order to provide timely and appropriate management [3–5].

## 2. Case 1

**Figure 1:**
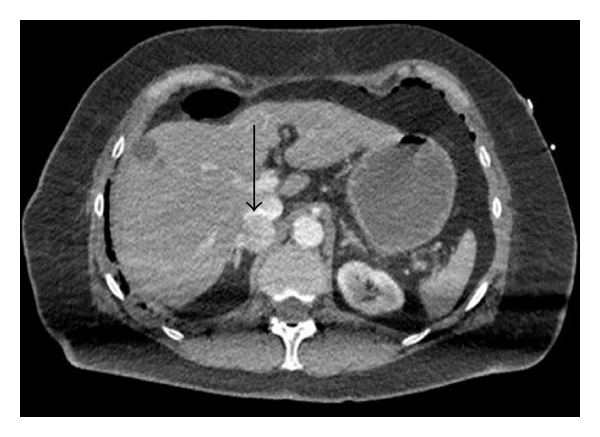
CT abdomen revealing a 22 mm nodule arising from the body of the right adrenal gland (arrow).

A 68-year-old female, underwent an elective laparoscopic cholecystectomy with an uneventful surgical and anaesthetic course. Her medical history included hypertension for 15 years and a previous surgery for breast carcinoma. Hypertension (BP 205/100 mmHg) was noted in the recovery ward which was partially resolved with opioid analgesia. The following morning she developed atrial fibrillation (rate 170 bpm), hypertension (BP 220/160 mmHg), pulmonary oedema, and lactic acidosis. She became progressively hypotensive and obtunded with a Glasgow coma scale of 4/15 and fixed dilated pupils, requiring intubation, mechanical ventilation, and inotropic vasopressor support with adrenaline and later noradrenaline infusions. Computed tomography scans of brain, chest, and abdomen were reported as unremarkable. Transthoracic echocardiogram revealed severe global left ventricular systolic impairment (ejection fraction <10%). She proceeded to coronary angiography which revealed normal coronary arteries and intra-aortic balloon counterpulsation was instituted. Reconsideration of the diagnosis prompted reevaluation of the radiological imaging, which revealed a 22 mm nodule arising from the body of the right adrenal gland (Figure 1). We therefore considered phaeochromocytoma to be the most likely underlying cause of the hypertensive crisis and subsequent cardiogenic shock. She remained profoundly hypotensive and we elected to add levosimendan 0.156 mcg/kg/min and vasopressin at 0.4 units/hour in order to minimise exogenous catecholamine use. A pulmonary artery catheter was inserted, which revealed low pulmonary artery occlusion pressure and cardiac output in keeping with hypovolaemia, and cautious fluid boluses were given. We considered further therapeutic options: emergent adrenalectomy under cardiopulmonary bypass, mechanical circulatory support with extracorporeal membrane oxygenation (ECMO), or left ventricular assist device (LVAD). In the event, the patient stabilised over the next 24 hours. Vasoactive support was discontinued on day 3, and a follow-up echocardiogram on day 4 revealed complete resolution of left ventricular dysfunction. On day four, alpha-blockade was instituted with commencement of phenoxybenzamine 10 mg twice daily by nasogastric tube. Due to concern regarding the absorption of phenoxybenzamine a phentolamine infusion was commenced at 5 mg/hr. Extubation and removal of the intra-aortic balloon pump occurred on day five. Serial 24 hr urine catecholamines collected on day six (72 hours after catecholamine infusions were discontinued) were elevated (noradrenaline 227 nmol/24 h [nr: 50–1600], adrenaline 850 nmol/24 h [nr: 5–122] and dopamine 388 nmol/24 h [nr: 300–3900]). Chromogranin A was 190 u/L (nr: 0–30). Plasma metanephrines performed on day seven were significantly elevated (normetanephrine 6596 pmol/L [nr: 120–1180], metanephrine 2094 pmol/L [nr: 80–510]). MIBG (metaiodobenzylguanidine) scanning showed focal uptake in relation to the right adrenal mass, supporting the diagnosis of phaeochromocytoma. The patient was discharged from intensive care on phenoxybenzamine 40 mg four times daily, nicardipine 20 mg three times daily, and metoprolol 150 mg once daily in preparation for adrenalectomy. Right laproscopic adrenalectomy was subsequently performed (six weeks from her initial surgery) without incident and histological examination confirming the diagnosis of a phaeochromocytoma. The patient made a full recovery.

## 3. Case 2

A 42-year-old female, with a history of borderline hypertension and type 2 diabetes mellitus, underwent elective endometrial ablation for definitive treatment of menorrhagia. While in the recovery room after an uneventful intraoperative course, she developed severe hypertension and pulmonary oedema with an ejection fraction of 25% on transthoracic echocardiograph. She was treated with nitrates and diuretics for a period of four days, following transfer to the local cardiac intensive care unit with clinical improvement. On further questioning, she reported a twelve-month history of palpitations, diaphoresis, and headache. Against this background and the development of a hypertensive crisis, 24 hr urinary catecholamines were measured and were found to be elevated (noradrenaline 5060 nmol/24 hr [nr: 50–1600], adrenaline 1349 nmol/24 h [nr: 5–122], and dopamine 1096 nmol/24 h [nr: 300–3900]). Chromogranin A was 240 u/L [nr: 0–30]. A CT abdomen revealed a 37 mm left adrenal mass, leading to a presumptive diagnosis of phaeochromocytoma and alpha-blockade was instituted with phenoxybenzamine 20 mg three times daily. She proceeded to an uneventful left laproscopic adrenalectomy eight weeks later, following which a phaeochromocytoma was confirmed histologically. Twenty-four-hour urine catecholamine levels normalised postoperatively. Genetic screening confirmed the phaeochromocytoma to be sporadic in origin and the patient made a full recovery.

## 4. Discussion

The differential diagnosis of hypertensive crisis is broad and may be divided into neurological, endocrine, and renal causes. Since profound hypertension of any cause may result in acute cardiac, renal, and neurological injury, identifying the primary event may be challenging, as in our first case in which the presentation with hypertension, tachyarrhythmia, cardiac failure, and coma clinically resembled catastrophic subarachnoid haemorrhage.

Cardiac manifestations of phaeochromocytoma include left ventricular hypertrophy and diastolic dysfunction, tachyarrhythmias, and systolic heart failure. In the setting of a hypertensive crisis, a tako-tsubo type, inverse tako-tsubo type, or an acute dilated cardiomyopathy can occur; systolic anterior motion with dynamic outflow obstruction or coronary artery spasm may lead to cardiogenic shock [6, 7]. Mechanisms underlying cardiogenic shock in phaeochromocytoma are related to the direct toxic effects of catecholamines on cardiac myocytes and coronary artery spasm with focal myocardial necrosis. Importantly, the reversibility of cardiac dysfunction in both chronic and acute presentations is well established [8–10] over timescales ranging from three weeks to a year. Our first patient had a relatively rapid recovery of left ventricular function, which appears to be one of the shortest reported recovery times from a phaeochromocytoma crisis. 

In our first patient, biochemical diagnosis was hindered by the use of adrenaline and noradrenaline infusions; therefore, urine catecholamine collections were postponed until 72 hours after these were discontinued. In addition, cardiogenic shock can physiologically increase catecholamine release. The definitive treatment for phaeochromocytoma is surgical resection, with adequate alpha-blockade (to prevent hypertensive cerebral events perioperatively), followed by beta-blockade if necessary [11] prior to surgery. The question arose in both cases as to the timing of adrenalectomy. Surgery in patients not adequately alpha-blocked is associated with increased mortality and morbidity [12]. A recent retrospective cohort study of patients presenting with phaeochromocytoma crisis found that stabilisation of the acute crisis, followed by alpha-blockade prior to elective was associated with a shorter hospital stay and fewer postoperative complications than emergency surgery [13]. Intensive care management of the patient with phaeochromocytoma crisis may be complex. In the hypertensive patient, with or without cardiac failure, the aims are to use careful titration of alpha-blockers and other agents to lower blood pressure over a 24–48-hour period, thus reducing left ventricular afterload and pathologic vasoconstriction, while avoiding too rapid a fall in cerebral perfusion pressure in patients whose autoregulatory threshold may be shifted rightwards. Patients in whom cardiogenic shock has supervened represent a greater challenge. Conventional management involves the use of inotropic agents such as dobutamine and adrenaline to maintain cardiac output, with vasopressors as required to treat hypotension. Although there is little evidence on which to base decisions, concern regarding receptor downregulation and potential for exacerbating the condition led us, in our first case, to use alternative agents as far as possible. Levosimendan, a calcium-sensitising agent with inodilator properties, appeared as a logical choice and has been used successfully in other patients [14]. Phosphodiesterase III inhibitors such as milrinone, and other novel inotropes such as glucagon and high-dose insulin could also have been employed. Due to profound hypotension, we added vasopressin as a noradrenaline-sparing agent.

Intravascular filling volumes are usually low as vasoconstriction is reduced with alpha-blockade; therefore, patients require careful fluid loading [15, 16]. In our first case, we were surprised to find a low pulmonary artery occlusion pressure in the presence of severe left ventricular dysfunction and pulmonary oedema, and this led us to give fluid boluses with significant haemodynamic improvement. We speculate, therefore, that the pulmonary oedema may have been partially due to endothelial injury and increased pulmonary vascular permeability rather than simply been cardiogenic in origin as we presumed initially. Measurements of extravascular lung water and pulmonary vascular permeability index would be helpful in future cases. Mechanical circulatory support has been used successfully. In our first case, we used intra-aortic balloon counterpulsation; however, others have used venoarterial ECMO and temporary LVADs with successful outcomes [17–19].

In summary, our two cases highlight the rare presentation of undiagnosed phaeochromocytoma presenting as “hypertensive crisis” and cardiogenic shock unmasked by elective nonadrenal surgical procedures. In both cases, successful elective adrenalectomy was performed after a period of 6–8 weeks after the hypertensive crisis. Phaeochromocytoma should be considered in the differential diagnosis of patients who develop a hypertensive crisis in the perioperative period.
